# Solid and Hollow Pre-Tensioned, Pre-Stressed Concrete Orchard Posts—Computational and Experimental Comparative Analysis

**DOI:** 10.3390/ma18112525

**Published:** 2025-05-27

**Authors:** Jarosław Michałek, Jacek Dudkiewicz

**Affiliations:** Faculty of Civil Engineering, Wrocław University of Science and Technology, Wyb. Wyspiańskiego 27, 50-370 Wrocław, Poland; jacek.dudkiewicz@pwr.edu.pl

**Keywords:** experimental research, orchard post, pre-tensioned, pre-stressed concrete

## Abstract

For several years now, fruit-growers have increasingly often used pre-tensioned, pre-stressed concrete posts for supporting branches of fruit trees and suspending protective nets in order to limit damage to fruits caused by hail, wind, snow, heavy rainfall, insects and birds. Pre-tensioned, pre-stressed concrete posts most often have a trapezoidal cross-section, which is ideally suitable for mass production in a self-supporting non-dismantlable steel mould on a pre-stressing bed. Posts with 70 mm × 75 mm, 80 mm × 85 mm and 90 mm × 95 mm cross-sections are typically produced, whereas 100 mm × 120 mm and 130 mm × 140 mm posts are manufactured to order. Furthermore, it is proposed to produce hollow posts. Such posts are lighter than solid posts, but they require a more complicated production technology. This paper presents selected parts of a comparative computational–experimental analysis of solid and hollow posts. In the Building Structures Laboratory in the Building Structures Department at the Civil Engineering Faculty of the Wrocław University of Science and Technology, experimental tests of pre-stressed concrete orchard posts of 70 mm × 75 mm and 90 mm × 95 mm with solid and hollow cross-sections were carried out on a full scale. The theoretical analysis and research has shown that the resistance to bending, cracking resistance and rigidity of hollow posts (with their cross-sectional outline unchanged) will not significantly differ from those of the currently produced solid posts. At same time, material savings will be achieved. Therefore, the main task is to master the continuous moulding of hollow posts from dense plastic concrete with the simultaneous pulling out of the cores, producing longitudinal hollows in the posts.

## 1. Introduction

**Figure 1:**
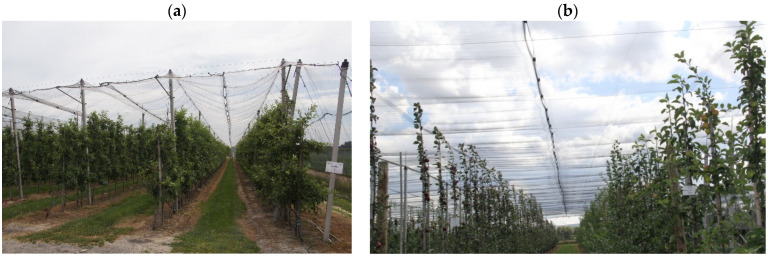
Nets on pre-tensioned, pre-stressed concrete posts: (**a**) conventional netting system (photo by J. Michałek); (**b**) flat netting system (photo by D. Łabanowska-Bury).

Fruit-bearing branches in orchards need to be supported by wires strung on 3.0 m-tall (above ground) wooden, steel or (reinforced or pre-tensioned, pre-stressed) concrete posts spaced at every 6–8 m [1,2,3]. In order to limit fruit losses in orchards, increasingly often, nets are mounted on such (but 4.0 m-high) posts (Figure 1), using the conventional, flat or cross netting system [4,5,6,7,8]. Nets are widely used to protect crops against hail, wind, snow or heavy rainfalls [9,10,11] in fruit farming and ornamentals cultivation, as well as for shading [12,13,14,15] and the slight modification of the microenvironment [16,17]. Nets are also used for protection against insects and birds [4,5,6,18,19,20].

In the conventional netting system [4,5,7,18], nets hang on ∅4 mm wires strung on posts located along rows of trees and spaced at every 3–4 m (Figure 1a). About 1 m below the top, there are strung cross wires stretching the nets downwards. In this way, a multi-bay “roof” with its planes inclined at 65° is formed, whereby hail balls can freely fall to the ground through the discharge holes in the place where the nets are joined together.

In the flat netting system [4,5,7,18], nets are stretched horizontally on posts and on orthogonally intersecting ∅5 mm wires (Figure 1b). On the posts, there are plastic caps with a wire lock and a screw for net fixing. At half the distance between the rows of trees, the nets are joined together with plastic clips. The length of the nets is equal to the length of the particular rows, while their width is 10–12% larger than the spacing of the rows of trees. The nets stretch under the weight of hail and subside in the interrows. Between the points where the nets are joined together with plastic clips, holes open, through which hail pours out beyond the tree crowns.

The cross-netting system [6,7] is a solution intermediate between the conventional system and the flat system. A horizontal surface protecting fruit trees against hail is formed by nets overlapping for 1.0–1.5 m. The nets are joined together with elastic ropes whereby during hail precipitation the surface of the nets in the interrows lowers uniformly. This netting system is the most expensive of the ones mentioned above, but it makes full orchard protection possible. Its short-life elements are the elastic ropes, which need to be replaced every 4–5 years.

**Figure 2:**
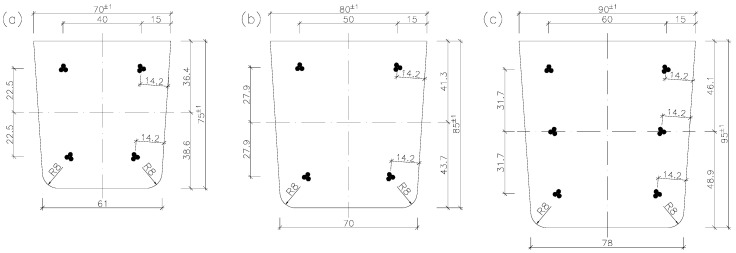
Cross-sections of typical orchard posts: (**a**) 70 mm × 75 mm, (**b**) 80 mm × 85 mm, (**c**) 90 mm × 95 mm.

Pre-tensioned, pre-stressed orchard posts are manufactured in the form of a 4.0 m (for orchards without nets) or 5.0 m (for orchards with nets) long-type series [7,21,22]. They are usually trapezoidal in cross-section (Figure 2). The internal (intermediate) posts in rows of trees are 70 mm × 75 mm (sometimes 70 mm × 80 mm) and 80 mm × 85 mm in cross-section (Figure 2a,b), while the edge posts (on the borders of plots) are 90 mm × 90 mm and 90 mm × 95 mm in cross-section (Figure 2c). The size range of the orchard posts shown in Figure 2 can be completed with bespoke 100 mm × 120 mm and 130 mm × 140 mm strong (end and corner) posts.

**Figure 3:**
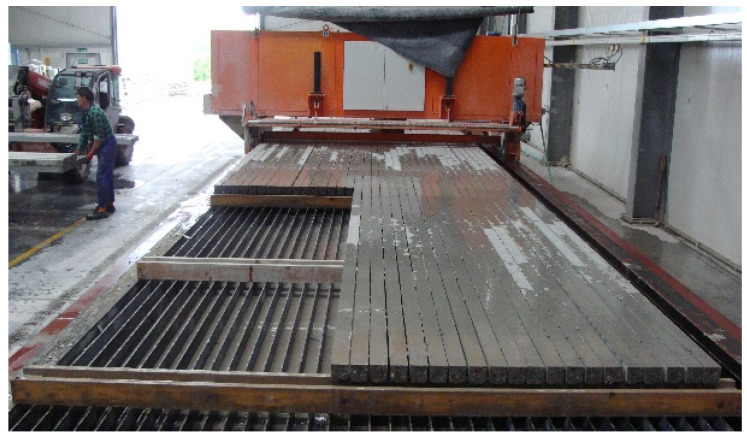
Cutting pre-tensioned strips into 4.0 or 5.0 m-long posts (photo by A. Łodo).

**Figure 4:**
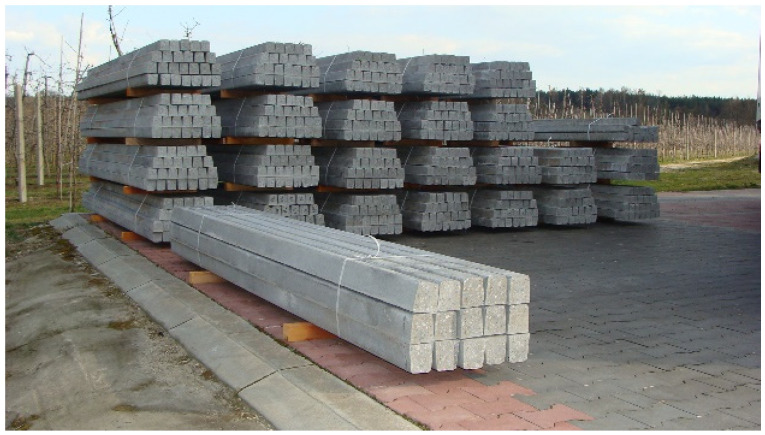
Ready posts fastened together into bundles in a storage yard (photo by A. Łodo).

Pre-tensioned, pre-stressed orchard posts are manufactured from concrete of C40/50 grade in a self-supporting battery moulds on a pre-stressing bed (Figure 3). The tendons are tensioned in groups by actuators located on the bed’s active side. Once the concrete reaches a technological strength of C35/45, the tension of the tendons is released and the bed-long elements are lifted above the mould’s trapezoidal troughs. The elements having the pre-stressing bed length, e.g., 64 m, are cut depending on the needs into sections agreed on with the customer (Figure 3). The ready posts with identical dimensions are fastened together into bundles (Figure 4). Using the above technology, 640 posts per day can be produced in one casting [21,23,24,25,26,27].

The posts are longitudinally reinforced with three-wire strands symmetrically located at the cross-section’s side walls (Figure 2). Tendons S3 made of ∅2.25 mm and ∅2.40 mm wire are particularly suitable for pre-stressing elements with small cross-sections [24]. The optimal number of tendons is four strands Y1860S3-4.9 in 70 mm × 75 mm posts and four strands Y1860 S3-5.2 (or Y2060 S3-5.2 [28]) in 80 mm × 85 mm posts (Figure 2a,b). No conventional reinforcement is used in the elements. Posts 80 mm × 85 mm in cross-section (Figure 2b) are produced in two pre-stressed versions (a basic version with pre-stress effected by four tendons, as in Figure 2b, to be used as through-posts, and a strengthened version with pre-stress effected by 6 tendons, to be used as end posts).

The thirty-year durability (class 1 according to the standard [29]) of pre-stressed concrete orchard posts operated under natural atmospheric environmental conditions (exposure class XC4 [30,31]—cyclically wet and dry environment, causing carbonation of the outer surface of the concrete and cyclic freezing/thawing of the concrete corresponding to exposure class XF1 [30,31]—vertical surfaces of concrete exposed to rain and freezing/thawing) is ensured by the characteristic compressive strength of concrete (minimum concrete class C40/50), minimum cover of pre-stressing reinforcement c_min_ = 10 mm, and maximum concrete absorption capacity of 6.5%. Since the soil and groundwater in the orchard area are not aggressive to concrete, there is no need for the mandatory coating of the girdle part of the posts with insulating agents at the stage of precast production or during their installation in the orchards.

**Figure 5:**
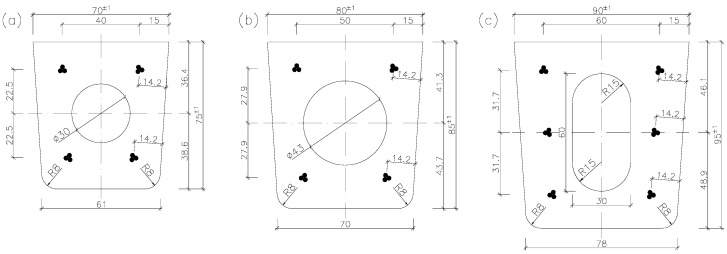
Cross-sections of pre-tensioned, pre-stressed concrete orchard posts with longitudinal hollows: (**a**) 70 mm × 75 mm, (**b**) 80 mm × 85 mm, (**c**) 90 mm × 95 mm.

Recently, a new proposal of producing longitudinally hollow posts (Figure 5) has appeared. The introduction of a hole in the cross-section does not generally cause a decrease in the load-bearing capacity and stiffness of the elements. It should be emphasized here that “slimming down” the element may have a wider impact not only on the decrease in the mass of the elements, but also on the reduction in material consumption, the reduction in transport costs, and consequently, direct and indirect reductions in CO_2_ emissions. Such posts are lighter than posts with a solid cross-section (Table 1), but their manufacturing technology is more complicated. Longitudinal hollows circular (Figure 5a,b) or oval (Figure 5c) in cross-section are simultaneously created in tens of posts by means of sliding cores pulled out of compacted concrete. Strands in hollow posts (Figure 5) should be located along the cross-section’s height as in solid posts (Figure 2) so that the resultant force coincides with the centroid of the hollow (the pre-stress should not result in the bending of the cross-section).

This paper presents the results of theoretical analyses and tests of two types of orchard posts with a 70 mm × 75 mm and 90 mm × 95 mm solid and hollow cross-sections, regarding their weight, resistance to bending, cracking resistance and rigidity.

## 2. Test Materials and Methods

**Figure 6:**
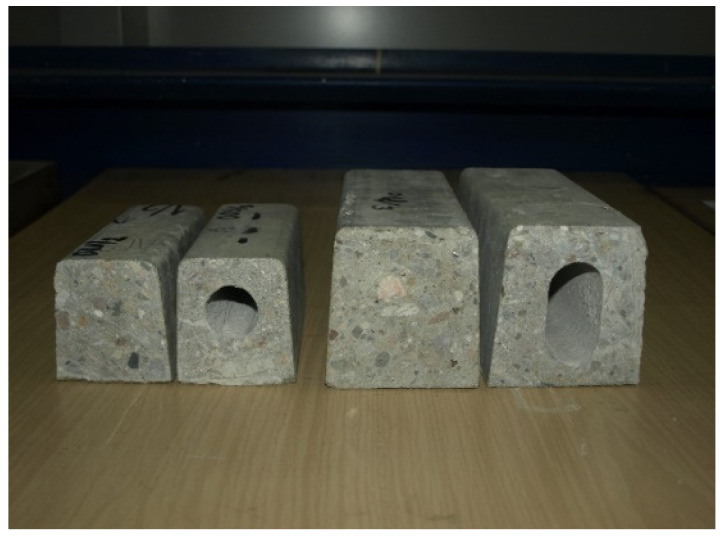
Cross-sections of tested orchard posts (photo by J. Michałek).

For the purpose of this research, 1.22 m-long pre-tensioned, pre-stressed concrete sections (Figure 6) cut from 70 mm × 75 mm and 90 mm × 95 mm orchard posts without a hollow (Figure 2a,c) and with a hollow (Figure 5a,c) were tested in the Building Structures Laboratory in the Building Structures Department at the Civil Engineering Faculty of the Wroclaw University of Science and Technology.

The posts were made of concrete of grade C40/50. The 70 mm × 75 mm posts were reinforced with four strands located symmetrically—one strand in each corner (Figure 2b and Figure 5b), while the 90 mm × 95 mm posts were reinforced with six strands located three at each of the cross-section’s side walls (Figure 2c and Figure 5c). In the solid posts three-wire strands of type Y1860 S3-4.9 were used, while in the hollow posts, strands of Y2060 S3-5.2 [28] were used. The distances of the strands from the side walls are the same for all the posts, each amounting to 14.2 mm. The average thickness of the nominal cover of the pre-stressing tendons from the top and bottom fibers amounts to about c_nom_ = 12 mm, which corresponds to the distance to the axis of the strands: a_p1_ = a_p2_ = 12 + 0.5 · 4.9 = 14.4 mm. The basic specifications of the strands are given in Table 1.

**Figure 7:**
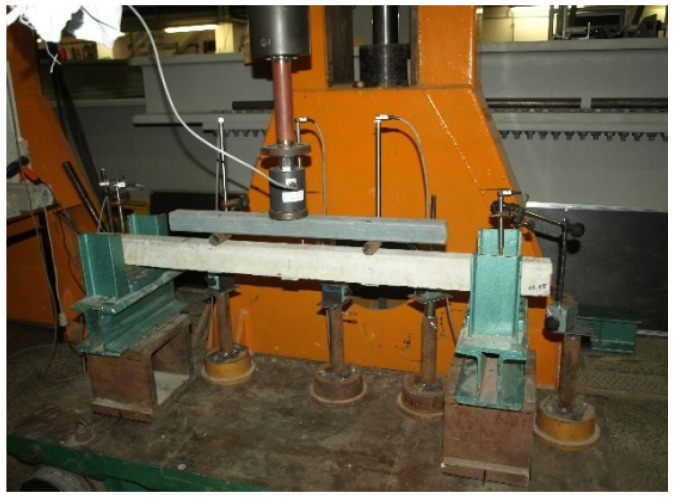
Stand for testing pre-tensioned, pre-stressed concrete orchard posts (photo by J. Michałek).

The sections of orchard posts delivered to the laboratory were subjected to geometry and weight measurements followed by strength tests on a special stand enabling testing in the horizontal position (Figure 7) as for a simply supported beam with a span of 1000 mm loaded with two-point forces at identical distances from the supports.

**Figure 8:**
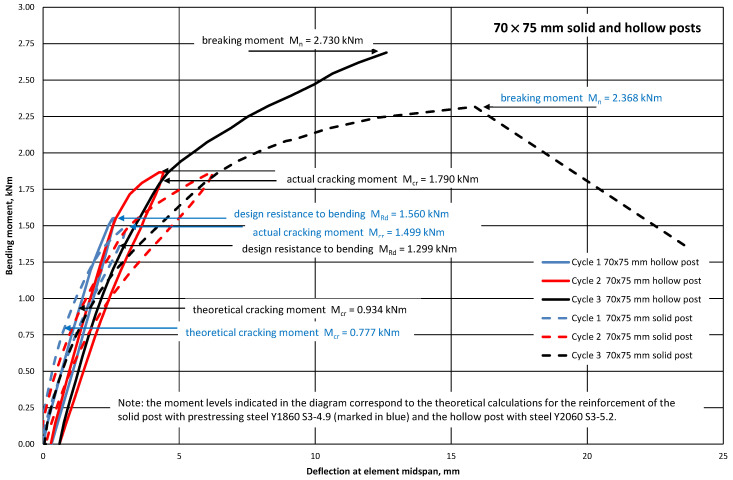
Deflection of 70 mm × 75 mm solid and hollow orchard posts versus bending moment at midspan L = 1.0 m.

**Figure 9:**
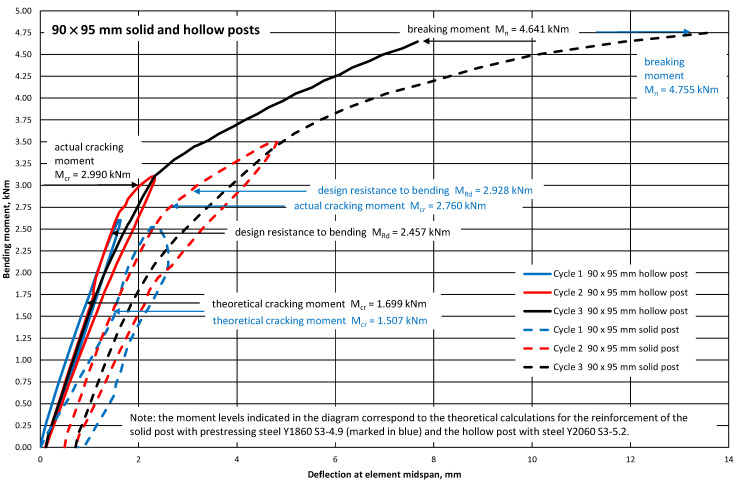
Deflection of 90 mm × 95 mm solid and hollow orchard posts versus bending moment at midspan L = 1.0 m.

The test stand consisted of two steel supports ensuring support for the 1200 mm-long tested elements 100 mm from their ends. The pre-tensioned, pre-stressed elements were subjected to loading by means of a first-accuracy-class Instron actuator, positioned in the middle of the element span, via a crosshead and two ∅30 mm steel rollers at a center-to-center spacing of 400 mm (Figure 7). The load was measured using a first-accuracy-class force gauge CL14 made by the Nonelectrical Quantities Measuring Electronics Plant in Marki. The force gauge can measure a force of up to 50 kN. This test setup made it possible to determine the values of the cracking moment and the breaking moment of the tested elements in a simple way [27]. During load application the deflections of the tested element under the forces and in the middle of its span were measured by a digital inductive displacement transducer WA100 with a measuring range of 0–100 mm and a resolution of 0.001 mm made by Hottinger Baldwin Messtechnik GMBW. The axial stiffness of the supports was monitored by means of WA10 sensors with a measuring range of 0–10 mm and a resolution of 0.001 mm, made by Hottinger Baldwin Messtechnik GMBW. During the tests, the instant of cracking and the development of the crack with increasing load were also controlled. The loading of the elements was conducted in accordance with the test plan shown in Figure 8 and Figure 9. First, the elements were loaded to the level of design load capacity M_Rd_, and then fully unloaded. In the next step, the elements were loaded to the level of calculated breaking moment M_n,t_ = 1.8 M_k_ and then fully unloaded. The final load cycle was conducted until failure caused by moment M_n_.

## 3. Results of the Theoretical Analyses and Their Discussion

The orchard posts were first subjected to computational analyses. The design strength of strand Y1860 S3-4.9 is f_pd_ = f_p0.2k_/γ_s_ = 1668/1.15 = 1450 MPa, and that of strand Y2060 S3-5.2 amounts to f_pd_ = 1831/1.15 = 1592 MPa. The initial force P_0_ in one ∅4.9 mm strand [28] is equal to P_01_ = P_01,eff_ = 13.14 kN (for ∅5.2 mm strands P_01_ = P_01,eff_ = 14.73 kN), which amounts to, respectively, 59.2% and 52.6% of force F_pk_ = 22.2 kN and force F_pk_ = 28.0 kN. The above values (P_01_ = 13.14 kN and P_01_ = 14.73 kN0 stem from the design assumptions for the pre-stressing beds used by the manufacturer of the posts [23].

According to standard [30], the final losses of pre-stress for stress σ_0,max_ = 0.6 · f_pk_ can be assumed to amount to 15%. The mean pre-stress force after the total losses in the ∅4.9 mm strand amounts to P_mt,1_ = 0.85 · P_01_ = 0.85 · 13.14 = 11.17 kN (in the ∅5.2 mm strand P_mt,1_ = 12.52 kN). The stresses in the strands after anchoring and taking into account the immediate pre-stress losses amount to σ_pm0.1_ = 0.95 · P_01_/A_p1_ = = 0.95 · 13.14 · 103/11.93 = 1046 MPa for the ∅4.9 mm strands and to σ_pm0.1_ = 0.95 · 14.73 · 103/13.60 = 1029 MPa for the ∅5.2 mm strands. The stresses after the total losses are taken into account amount to σ_pmt,1_ = P_mt,1_/A_p1_ = 11.17·103/11.93 = 936 MPa in the ∅4.9 mm strands and to σ_pmt,1_ = 12.52 · 103/13.60 = 921 MPa in the ∅5.2 mm strands, which means that they constitute, respectively, 50.3% and 44.7% of strength f_pk_ = 1860 MPa and strength f_pk_ = 2060 MPa. For the above assumptions, the basic technical parameters of the analyzed orchard posts were determined (Table 2).

It follows from the calculations of the nominal unit weight of the 70 mm × 75 mm post with a ∅30 mm hollow that it is 14.8% lighter than the nominal weight of the 70 mm × 75 mm post without a hollow. The nominal unit weight of the 80 mm × 85 mm post with a ∅43 mm hollow is identical to that of the 70 mm × 75 mm post (the ∅43 mm diameter hollow in the 80 mm × 85 mm post was deliberately selected in order to equalize the weights). Relative to the nominal weight of the 80 mm × 85 mm solid post, the ∅43 mm hollow reduced the weight of the hollow post by 23.3%. The oval hollow in the cross-section of the 90 mm × 95 mm post reduced the latter’s weight by 20.1%. The weight of the 90 mm × 95 mm post with a cross-section with an oval hollow became equal to the weight of the 80 mm × 85 mm post without a hollow. This means that one can manufacture pre-tensioned, pre-stressed concrete posts with 80 mm × 85 mm and 90 mm × 95 mm cross-sections with a longitudinal hollow, whose weights of 1 linear meter will be respectively equal to those of the 70 mm × 75 mm and 80 mm × 85 mm solid posts (Table 2), but which will have better strength characteristics.

It appears from Table 2 that the theoretical resistance to bending M_Rd2_ = 1.299 kNm of the 70 mm × 75 mm posts with the 4∅5.2 mm reinforcement (A_p_ = 4 × 13.60 mm^2^) is lower by about 16.7% than the resistance M_Rd1_ = 1.560 kNm of the post with the 4∅4.9 mm reinforcement (A_p_ = 4 × 11.93 mm^2^), despite the fact that the cross-sections of the ∅5.2 mm strands are larger (by about 14%) than those of the ∅4.9 mm strands. The 70 mm × 75 mm posts made of concrete of strength class C40/50 should not be pre-stressed with strands stronger than 4∅4.9 mm. The introduction of a ∅30 mm hollow into the centroid of the cross-section of the 70 mm × 75 mm posts pre-stressed with 4∅4.9 mm strands will not result in a decrease in resistance to bending M_Rd_ in comparison with the solid cross-section (Table 2). The use of four Y2060 S3-5.2 strands for reinforcing solid or hollow 70 mm × 75 mm posts is not economically viable, as it reduces the calculated theoretical resistance to bending (M_Rd2_ = 1.299 kNm) by 16.7% in comparison with the resistance M_Rd1_ = 1.560 kNm of the posts reinforced with steel Y1860 S3-4.9. This conclusion holds true for concrete grade C40/50 and the use of the same elongation for ∅4.9 mm and ∅5.2 mm strands [24].

The 80 × 85 mm solid posts originally were to be pre-stressed with six Y1860 S3-4.9 tendons located symmetrically on both sides of the cross-section (similarly as in the case of the 90 mm × 95 mm posts—Figure 2c). Because of the great interest in the stronger 80 × 85 mm posts (however, these were too expensive for through-posts with 6∅4.9 mm reinforcement), a decision was made to manufacture 80 mm × 85 mm posts [23,24] with 4∅4.9 mm (alternatively 4∅5.2 mm) reinforcement. The use of 4∅4.9 mm tendons for pre-stressing 80 mm × 85 mm posts, which are 30% heavier than the 70 mm × 75 mm posts (Table 2), turned out to be profitable, as the design resistance M_Rd_ of the 80 mm × 85 mm solid posts is 30% higher than the resistance of the 70 mm × 75 mm posts with the same 4∅4.9 mm reinforcement. The replacement of 4∅4.9 mm tendons with 4∅5.2 mm tendons results in a further increase (by 13.7%) in the bending resistance of the 80 mm × 85 mm posts relative to the 70 mm × 75 mm posts with the 4∅4.9 mm reinforcement. The use of four Y2060 S3-5.2 tendons for reinforcing the 80 mm × 85 mm hollow post results in an increase in its resistance to bending M_Rd2_ = 2.202 kNm by 10.1% in comparison with the resistance M_Rd1_ = 2.040 kNm of the post reinforced with four Y1860 S3-4.9 tendons (Table 2). The theoretical resistance M_Rd2_ = 2.202 kNm of the 80 mm× 85 mm hollow post pre-stressed with the 4∅5.2 mm reinforcement is, however, lower by 5.1% than the resistance M_Rd2_ = 2.320 kNm of the solid post with the same 4∅5.2 mm pre-stress.

The resistance to bending M_Rd_ of the 90 mm × 95 mm solid post is almost identical for six tendons Y1860 S3-4.9 and Y2060 S3-5.2, and amounts to about 2.9 kNm (Table 2). The resistance to bending (M_Rd1_ = 2.832 kNm) of the 90 mm × 95 mm post with an oval hollow, reinforced with steel Y1860 S3-4.9, is 3.3% lower than the resistance (M_Rd1_ = 2.928 kNm) of the solid post. The use of pre-stressing steel Y2060 S3-5.2 in the 90 mm × 95 mm hollow post results in a decrease in its resistance (M_Rd_ = 2.457 kNm) by 13.2% relative to the resistance (M_Rd_ = 2.832 kNm) of the post pre-stressed with steel Y1860 S3-4.9. This is due to the over-reinforcement of the hollow post.

Cracking moments M_cr_ for pre-tensioned, pre-stressed concrete posts with cross-sections as shown in Figure 2 and Figure 5 were determined for rectangular cross-sectional outlines. The use of a longitudinal hollow with diameter D = 30 mm in the pre-stressed 70 mm × 75 mm posts (Figure 5a) results in an increase in cracking moment by 10.2% when pre-stressing with tendons Y1860 S3-4.9 and by 10.5% when pre-stressing with tendons Y2060 S3-5.2 (Table 2). In the 80 mm × 85 mm posts, a hollow with diameter D = 43 mm (Figure 5b) results in an increase in cracking moment by 14.5% for pre-stressing with tendons Y1860 S3-4.9 and by 15.3% for pre-stressing with tendons Y2060 S3-5.2 (Table 2). The use of an oval hollow in the 90 mm × 95 mm posts (Figure 5c) results in an increase in cracking moment by 3.4% when pre-stressing with tendons Y1860 S3-4.9 and by 3.9% when pre-stressing with tendons Y2060 S3-5.2 (Table 2).

The deflection of an element depends on the load diagram, the values of the loads acting on the element and their duration, and on the span of the element and its stiffness in the elastic phase or in the cracked phase. Because of the diverse load diagrams and load values, only a description of the rigidity of the element’s cross-section in the uncracked phase (B_I_ = E_cm_ · I_c_) was used to evaluate the deflections of the pre-tensioned, pre-stressed concrete orchard posts. Concrete of strength class C40/50 (E_cm_ = 35 GPa) was assumed in the calculations. A longitudinal hollow in the cross-section of the 70 mm × 75 mm, 80 mm × 85 mm and 90 mm × 95 mm orchard posts in each of the cases causes a slight decrease in the second moment of area (I_c_), and consequently an increase in element deflection in comparison with the post with a solid cross-section. The introduction of an oval hollow into the cross-section of the 90 mm × 95 mm post results in a decrease in the second moment of area by 14% (Table 2) in comparison with the solid posts (whereby deflection increases by 16.3%). The smallest decrease in second moment of area (just 1.8%) occurs in the 70 mm × 75 mm post with a hollowed cross-section (Table 2). In the 80 mm × 85 mm post, the decrease in I_c_ amounts to 4.6% (Table 2). The increase in the deflection of the hollow elements in comparison with the solid ones is inversely proportional to the decrease in second moment of area (e.g., in the hollow 80 mm × 85 mm posts, a decrease in I_c_ by 4.6% causes an increase in deflection by 4.8%).

## 4. Test Results and Their Discussion

The experimental tests carried out in the Building Structures Laboratory in the Building Structures Department at the Civil Engineering Faculty of the Wroclaw University of Science and Technology were to serve as a verification of theoretical calculations. Hence, the research sample is not large, and includes one element from each type of orchard post analyzed. The tests were carried out on elements in a natural scale with a limited length of 1.22 m.

Measurements of the actual weight show that the theoretical unit weights of the pre-tensioned, pre-stressed concrete orchard posts (Table 2) corresponded to the values determined by weighing the precast elements. The weighing results indicate that the weight by volume of the concrete used for the production of the pre-tensioned precast concrete posts is γ_c_ ≅ 2500 kg/m^3^.

The values of cracking moment M_cr_ and design bending resistance M_Rd_ (as well as characteristic moment M_k_ = M_Rd_/γ_Q_ = M_Rd_/1.5) yielded by the theoretical analysis for the particular types of posts were used in the load tests of the pre-tensioned, pre-stressed concrete posts.

Figure 8 and Figure 9 show graphs of deflections of the tested pre-tensioned, pre-stressed concrete elements (70 mm × 75 mm and 90 mm × 95 mm solid and hollow posts) represented by a 1.0 m-long, simply supported beam loaded in its span with two forces at a distance of 0.3 m from the supports. During the tests, it was found that the elements did not undergo cracking in the operational phase (the test loads were equal to the design resistance to bending M_Rd_). The bending stiffnesses of the tested 70 mm × 75 mm elements (with hollow and solid cross-sections) in the elastic phase were similar (Figure 8). However, the bending stiffnesses of the tested 90 mm × 95 mm elements with hollow and solid cross-sections in the elastic phase differed (Figure 9). The occurrence of elongated holes in the cross-sections of orchard posts, 70 mm × 75 mm and 90 mm × 95 mm in each case, caused a decrease in the moment of inertia of the cross-section I_c_ (the highest for posts 90 mm × 95 mm—Table 2), and consequently an increase in the deflection of the element in relation to the post with a solid cross-section (Figure 8 and Figure 9). Additionally, the differences in the course of the graphs visible in Figure 9 may result from many other phenomena, e.g., the age of the tested elements, the actual value of the pre-stressing force, and the actual material parameters. The breaking moments M_n_ yielded by the tests of the solid posts pre-stressed with ∅4.9 mm tendons and the hollow posts with ∅5.2 mm pre-stress are almost identical (Table 3), despite the larger (by 14.0%) cross-sections of the ∅5.2 mm tendons in comparison with those of the ∅4.9 mm tendons.

**Figure 10:**
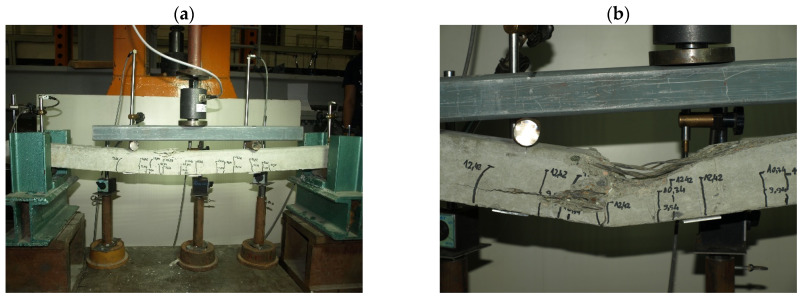
Failure of 70 mm × 75 mm solid orchard post as result of concrete compressed zone crushing: (**a**) general view of the destroyed element, (**b**) close-up of the destruction zone (photo by J. Michałek).

**Figure 11:**
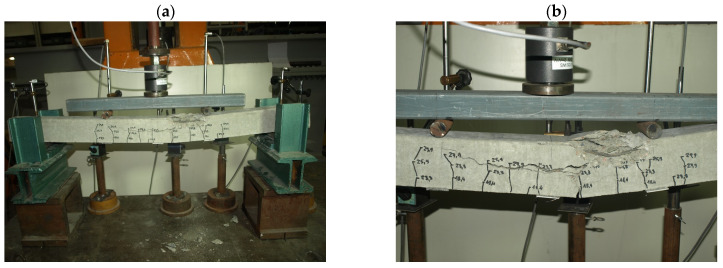
Failure of 90 mm × 95 mm solid orchard post as result of concrete compressed zone crushing: (**a**) general view of the destroyed element, (**b**) close-up of the destruction zone (photo by J. Michałek).

**Figure 12:**
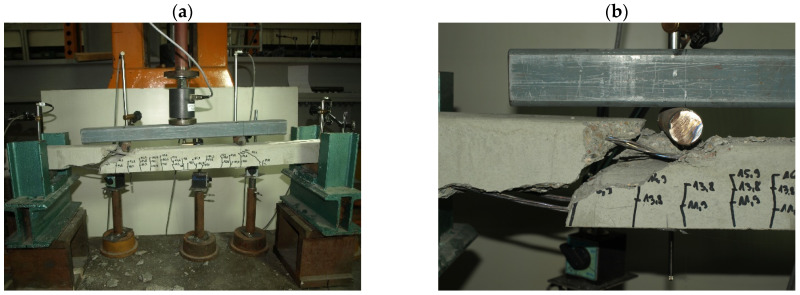
Failure of 70 mm × 75 mm hollow orchard post in zone of combined bending and shearing: (**a**) general view of the destroyed element, (**b**) close-up of the destruction zone (photo by J. Michałek).

**Figure 13:**
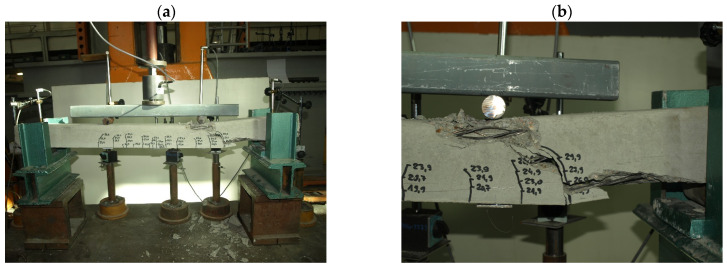
Failure of 90 mm × 95 mm hollow orchard post in zone of combined bending and shearing: (**a**) general view of the destroyed element, (**b**) close-up of the destruction zone (photo by J. Michałek).

Characteristic moments M_k_ = M_Rd_/γ_Q_ = M_Rd_/1.5 (M_Rd_—the theoretical resistance to bending of the cross-section and γ_Q_—a wind load coefficient) were introduced into the analysis of the test results to assess the bending resistance of the tested elements (posts) by determining the global safety factor for failure in bending: s = M_n_/M_k_ ≥ s_min_. The minimum value of the bending failure safety factor for the pre-tensioned, pre-stressed concrete orchard posts was assumed to amount to s_min_ = 2.2 [32] (Table 3). The solid pre-tensioned, pre-stressed concrete elements failed as a result of bending in the span (Figure 10 and Figure 11). The failure (Figure 12 and Figure 13) of the hollow pre-tensioned, pre-stressed concrete elements occurred in the zone of the combined action of bending and shearing (such a case of failure will not occur in orchard posts as they behave as long cantilevers).

It appears from the results presented in Table 3 that the introduction of a longitudinal hollow into the cross-section of the posts with dimensions as shown in Figure 5 does not result in a significant reduction in design resistance to bending M_Rd_, but contributes to an increase in cracking moment M_cr_. The calculation results were fully corroborated by experiments. The bending resistance tests of the solid and hollow pre-tensioned, pre-stressed concrete orchard posts showed their very good elastic performance and proper bending rigidity. The concrete in the tested elements is characterized by high cracking resistance and strength, as evidenced by the values of M_cr_, M_n_ and s (Table 3) obtained from the tests. It is advisable to conduct experimental studies in the future on a larger sample of elements, which will allow for a more statistical presentation of the research results.

## 5. Conclusions

For several years, string concrete orchard posts with full trapezoidal cross-sections and dimensions shown in Figure 1 have been manufactured in Poland. A supplement to the type with the series of dimensions shown in Figure 2 are posts with cross-sections of 100 mm × 120 mm and 130 mm × 140 mm. Recently, a new product has appeared on the Polish market in the form of lighter-than-usual posts with a longitudinal hole, the cross-sections of which are shown in Figure 5. Concrete posts with a longitudinal hole (i.e., hollow posts) are manufactured similarly to solid posts.

The calculations show the nominal weight of 1 linear meter of the smallest (70 mm × 75 mm) posts with a ∅30 mm hollow in their cross-section to be 14.8% lighter than that of the posts with a solid cross-section. In the case of the 90 mm × 95 mm post with an oval hollow in its cross-section, this weight is 20.1% lighter in comparison with the post with a solid cross-section.

For the same pre-stress and concrete strength class, the use of hollow cross-sections (Figure 5) in pre-tensioned, pre-stressed concrete orchard posts only slightly reduces their design resistance to bending M_Rd_ relative to the solid posts (Figure 2). For reinforcing 70 mm × 75 mm and 90 mm × 95 mm posts, it is enough to use pre-stressing reinforcement Y1860 S3-4.9. In the case of 80 mm × 85 mm posts pre-stressed with four tendons, it is worth using the stronger Y2060 S3-5.2 tendons (Table 2).

For the hollow posts, the cracking moment was found to be 8-10% higher than for the solid posts. A longitudinal hollow in orchard posts results in a slight decrease in second moment of area I_c_. Consequently, the deflections of hollow elements should slightly increase in comparison with solid elements (but this has no major bearing on the performance characteristics of orchard posts).

The strength tests of the 70 mm × 75 mm and 90 mm × 95 mm solid and hollow pre-tensioned, pre-stressed concrete posts showed their very good elastic properties, manifesting themselves in a return to the original shape after overloading by 80% relative to the service load. The posts show high cracking resistance and low permanent set values after overload. The tested sections of 70 mm × 75 mm and 90 mm × 95 mm solid and hollow pre-tensioned, pre-stressed concrete posts were characterized by a safety factor for failure in bending (s) above the minimum value s_min_ = 2.2 assumed for pre-tensioned, pre-stressed concrete elements with a decisive wind load.

The theoretical analyses and the tests have shown that the use of a cross-section with a hollow (with no change in the cross-sectional outline—Figure 2 and Figure 5) will not significantly affect the bending resistance, cracking resistance and rigidity of such posts (Figure 5) in comparison with the currently produced solid orchard posts (Figure 2). The benefit is the reduced weight of the posts. It should be emphasized here that “slimming down” the element can have a wider impact not only on the weight of the elements, but also in terms of the reduction in material consumption, the reduction in transport costs, and the need for smaller devices for driving posts into the ground. These are important issues in the context of sustainable development goals, such as lower CO_2_ emissions, and reducing the impact on the environment (noise and vibrations during the installation process).

The greatest economic effects from the introduction of longitudinally hollow pre-tensioned, pre-stressed concrete posts (Figure 5) onto the market can be brought about by replacing solid posts (e.g., 70 mm × 75 mm ones with a pre-stress of 4∅4.9 mm—Figure 2a) with hollow posts (e.g., 80 mm × 85 mm ones with an identical pre-stress of 4∅4.9 mm—Figure 5b and the same unit weight m = 12.2 kg/m as in the case of the 70 mm × 75 mm solid post—Table 2). No reduction in material (concrete and pre-stressing steel) consumption is obtained in this way, but the in-service resistance to bending M_Rd_ = 2.040 kNm (Table 2) of the 80 mm × 85 mm posts with a ∅43 mm hollow (Figure 5b) is 30.8% higher than the resistance M_Rd_ = 1.560 kNm (Table 2) of the 70 mm × 75 mm solid post.

A change in pre-stress in the 80 mm × 85 mm hollow post from 4∅4.9 mm to 4∅5.2 mm will result in an increase in its design resistance to bending up to M_Rd_ = 2.202 kNm, i.e., it will be 7.9% higher than the resistance M_Rd_ = 2.040 kNm of the post with the pre-stress of 4∅4.9 mm, but as much as 41.2% higher than the resistance M_Rd_ = 1.560 kNm of the 70 mm × 75 mm post. Therefore, it is worth using ∅5.2 mm pre-stressing steel in 80 mm × 85 mm posts, since savings in the number of posts installed in an orchard can be made (e.g., the spacing of 80 mm × 85 mm hollow posts can be increased by 30 or 40% in comparison with 70 mm × 75 mm solid posts).

The cost-effective mass production of hollow pre-tensioned, pre-stressed concrete posts (Figure 5) on a long pre-stressing bed (such as the one used for the manufacture of solid posts—Figure 2) is possible once the technology of the continuous moulding of hollow posts from dense plastic concrete with the simultaneous pulling out of the cores, creating longitudinal hollows in the posts, is mastered. Although pre-tensioned, pre-stressed posts with trapezoidal cross-sections with a longitudinal hollow (as in Figure 5) can be relatively easily made in laboratory conditions, the technology of the mass production of hollow pre-tensioned, pre-stressed concrete posts on a long pre-stressing bed enabling the casting of tens of elements along the width of the battery mould is undoubtedly a novelty. Mastering such production is a challenge, especially with the small cross-sections we are dealing with here. It is necessary to ensure that the strands in hollow cross-section posts are distributed at the height of the cross-section as in solid cross-section posts. The resultant force in the tendons must coincide with the center of gravity of the opening (the pre-stressing should not cause bending of the cross-section). Additionally, for reasons of durability and load-bearing capacity, the position of the core forming the opening in the cross-section and the proper thicknesses of the cross-section walls must be maintained. Work on the appropriate technology is still in progress and it is not yet possible to speak of commercial success.

## Figures and Tables

**Table 1:** Technical specifications of three-wire strands used for pre-stressing orchard posts [28].

Strand Designation	Y1860 S3-4.9	Y2060 S3-5.2
Wire diameter ∅ [mm]	2.25	2.40
Nominal strand diameter ∅_nom_ [mm]	4.90	5.20
Strand cross-sectional area A_p_ [mm^2^]	11.93	13.60
Strand weight [g/m]	94.0	106.2
Characteristic force F_pk_ = F_mk_ [kN]	22.200	28.000
Characteristic strength f_pk_ = R_m_ = F_pk_/A_p_ [MPa]	1861	2059
Force corresponding to yield point F_p0.1k_ (F_p0.2k_) [kN]	(19.900)	24.900
Yield point f_p0. 1k _(f_p0.2k_) [MPa]	(1668)	1831
Total elongation ε_uk_ [%] at rupture	≥3.5	≥3.5
Elastic modulus E_p_ [MPa]	196,000	195,000

**Table 2:** Comparison of nominal weight m, design resistance to bending M_Rd_, cracking moment M_cr_ and second moment of area (I_c_) in pre-tensioned, pre-stressed orchard posts.

Properties	Cross-Sections of Pre-Tensioned, Pre-Stressed Orchard Posts					
	70 mm × 75 mm		80 mm × 85 mm		90 mm × 95 mm	
	Solid (Figure 2a)	Hollow (Figure 5a)	Solid (Figure 2b)	Hollow (Figure 5b)	Solid (Figure 2c)	Hollow (Figure 5c)
Nominal unit weight m [kg/m]	12.2	10.4	15.9	12.2	19.9	15.9
Resistance to bending M_Rd_ [kNm]	1.560 (1.299)	1.560 (1.299)	2.040 (2.320)	2.040 (2.202)	2.928 (2.863)	2.832 (2.457)
Cracking moment M_cr_ [kNm]	0.777 (0.845)	0.856 (0.934)	0.952 (1.029)	1.090 (1.186)	1.507 (1.636)	1.558 (1.699)
Second moment of area I_c_ [m^4^]	2.303 × 10^−6^	2.263 × 10^−6^	3.838 × 10^−6^	3.670 × 10^−6^	6.002 × 10^−6^	5.263 × 10^−6^

The basic way of reinforcing the posts is by means of pre-stressing strands Y1860 S3-4.9. The results of calculations for strands Y2060 S3-5.2 are given in brackets. The strands are located as in Figure 2 and Figure 5.

**Table 3:** Design and experimental bending moments of orchard posts made of C40/50 concrete.

Post Cross-Section	Cracking Moment M_cr_		Design Bending Resistance M_Rd_	Characteristic Moment M_k_	Breaking Moment M_n_	s = M_n_/M_k_ (s > s_min_ = 2.2)
	Theoretical	Experimental				
	kNm					
solid 70 mm × 75 mm	0.777 (Y1860 S3-4.9)	1.499	1.560	1.040	2.368	2.28
hollow 70 mm × 75 mm	0.934 (Y2060 S3-5.2)	1.790	1.299 (1.56 for C50/60)	0.866 (1.04 for C50/60)	2.730	3.15 (2.63 for C50/60)
solid 90 mm × 95 mm	1.507 (Y1860 S3-4.9)	2.760	2.928	1.952	4.755	2.44
hollow 90 mm × 95 mm	1.699 (Y2060 S3-5.2)	2.990	2.457 (2.98 for C50/60)	1.638 (1.99 for C50/60)	4.641	2.83 (2.33 for C50/60)

The results in brackets are for over-reinforced posts made of C50/60 concrete, whose bending resistance M_Rd_ will be higher by about 20% than the theoretical resistance of posts made of C40/50 concrete.

## Data Availability

The original contributions presented in this study are included in the article. Further inquiries can be directed to the corresponding author.

## References

[1] Wasiak A. (2011). Prestressed concrete posts—Solid construction. Sad Nowocz..

[2] Wasiak A. (2011). Anti-hail nets on wood or concrete?. Sad Nowocz..

[3] Werner T. (2011). Prestressed concrete plants. Sad Nowocz..

[4] Castellano S., Mugnozza G.S., Russo G., Briassoulis D., Mistriotis A., Hemming S., Waaijenberg D. (2008). Plastics Net in Agriculture: A General Review of Types and Applications. Appl. Eng. Agric..

[5] Castellano S., Mugnozza G.S., Russo G., Briassoulis D., Mistriotis A., Hemming S. (2008). Design and use criteria of netting systems for agricultural production in Italy. J. Agric. Eng..

[6] Krupa T. (2012). A few words about nets and foils. Sad Nowocz..

[7] Kubiak J., Łodo A., Michałek J. (2014). Pre-tensioned prestressed concrete posts for fruit culture. Mater. Bud..

[8] Manja K., Aoun M. (2019). The use of nets for tree fruit crops and their impact on the production: A review. Sci. Hortic..

[9] Kiprijanovski M., Gjamovski V., Arsov T. (2015). The effects of anti-hail net in protection of pear orchard after hailstorm occurrence. Acta Hortic..

[10] Amarante C.V.T., Steffens C.A., Argenta L.C. (2010). Radiation, yield, and fruit quality of ‘gala’ apples grown under white hail protection nets. Acta Hortic..

[11] Guo J., Guo Y., Tong P., Wang X., Wang J. (2025). Effects of Different Coverage Years of Hail-Proof Nets on Environment, Leaf Traits and Fruit Quality in Apple Orchards. Horticulturae.

[12] Milivojević J., Radivojević D., Djekić I., Spasojević S., Dragišić Maksimović J., Milosavljević D., Maksimović V. (2025). Differentially Colored Photoselective Nets as a Sophisticated Approach to Improve the Agronomic and Fruit Quality Traits of Potted Blueberries. Agronomy.

[13] Zhang Y., Chu B., Zhang D., Li Q., Li Q., Li X., Liu Z., Ma F., Guan Q., Zhang D. (2023). Effects of Four Photo-Selective Colored Hail Nets on an Apple in Loess Plateau, China. Horticulturae.

[14] Bastías R.M., Manfrini L., Grappadelli L.C. (2012). Exploring the potential use of photo-selective nets for fruit growth regulation in apple. Chil. J. Agric. Res..

[15] Brglez Sever M., Tojnko S., Unuk T. (2015). Impact of various types of anti-hail nets on light exposure in orchards and quality parameters of apples–a review. Agricultura.

[16] Treder W., Mika A., Buler Z., Klamkowski K. (2016). Effects of hail nets on orchard light microclimate, apple tree growth, fruiting and fruit quality. Acta Sci. Pol. Hortorum Cultus.

[17] Girona J., Behboudian M.H., Mata M., Del Campo J., Marsal J. (2012). Effect of hail nets on the microclimate, irrigation requirements, tree growth, and fruit yield of peach orchards in Catalonia (Spain). J. Hortic. Sci. Biotechnol..

[18] Castellano S., Starace G., De Pascalis L., Lippolis M., Mugnozza G.S. (2016). Test results and empirical correlations to account for air permeability of agricultural nets. Biosyst. Eng..

[19] Nelson S.G.A., Klodd A.E., Hutchison W.D. (2023). Hail netting excludes key insect pests and protects from fruit damage in a commercial Minnesota apple orchard. J. Econ. Entomol..

[20] Chouinard G., Pelletier F., Larose M., Knoch S., Pouchet C., Dumont M.J., Tavares J.R. (2022). Insect netting: Effect of mesh size and shape on exclusion of some fruit pests and natural enemies under laboratory and orchard conditions. J. Pest Sci..

[21] https://www.spinazzegroup.com/en/concrete-poles-for-orchards/.

[22] https://www.comavit.it/en/concrete-posts-for-vineyards-orchards/.

[23] Łodo A., Kozioł P., Organek P. (2014). Form for the production of prestressed concrete posts for orchard plantations. Mater. Bud..

[24] Michałek J., Łodo A. Production technology of small-size pre-tensioned prestressed concrete elements. Proceedings of the Scientific and Technical Conference: Prestressed Structures.

[25] https://www.goodfruit.com/building-local-supports-for-concrete-trellis-posts/.

[26] https://www.contec-italy.eu/products/green-experience-for-vineyards-and-orchards/.

[27] Bayat H., Chalecki M., Lesniewska A., Maj M., Rybak J., Ubysz A. (2024). The cyclic load effect on the elasticity and plasticity deformation of high-strength reinforced concrete elements. Arch. Civ. Mech. Eng..

[28] (2011). Prestressing Steel. Part 3: Strand.

[29] (2012). Precast Concrete Products. Elements for Fences.

[30] (2004). Eurocode 2: Design of Concrete Structures. Part 1-1: General Rules and Rules for Buildings.

[31] (2023). Common Rules for Precast Concrete Products.

[32] (1957). Prestressed Concrete Structures. Design Rules.

